# Pharmacogenomics of Antibiotics

**DOI:** 10.3390/ijms21175975

**Published:** 2020-08-19

**Authors:** Gabriele Stocco, Marianna Lucafò, Giuliana Decorti

**Affiliations:** 1 Department of Life Sciences, University of Trieste, I-34128 Trieste, Italy; stoccog@units.it; 2 Institute for Maternal and Child Health—IRCCS “Burlo Garofolo”, I-34137 Trieste, Italy; marianna.lucafo@burlo.trieste.it; 3 Department of Medicine, Surgery and Health Sciences, University of Trieste, I-34149 Trieste, Italy

**Keywords:** pharmacogenomics, antibiotics, human leukocyte antigen (HLA), adverse drug reaction, transporters, pharmacokinetics

## Abstract

Although the introduction of antibiotics in medicine has resulted in one of the most successful events and in a major breakthrough to reduce morbidity and mortality caused by infectious disease, response to these agents is not always predictable, leading to differences in their efficacy, and sometimes to the occurrence of adverse effects. Genetic variability, resulting in differences in the pharmacokinetics and pharmacodynamics of antibiotics, is often involved in the variable response, of particular importance are polymorphisms in genes encoding for drug metabolizing enzymes and membrane transporters. In addition, variations in the human leukocyte antigen (HLA) class I and class II genes have been associated with different immune mediated reactions induced by antibiotics. In recent years, the importance of pharmacogenetics in the personalization of therapies has been recognized in various clinical fields, although not clearly in the context of antibiotic therapy. In this review, we make an overview of antibiotic pharmacogenomics and of its potential role in optimizing drug therapy and reducing adverse reactions.

## 1. Introduction

Since the introduction of sulfonamides in 1933 [1], and some years later of penicillin G [2], a large number of antibiotics have been introduced into clinics for the prevention and therapy of bacterial infections. These agents have completely changed the history of infectious disease, dramatically reducing their morbidity and mortality. Response to antimicrobial agents is however often variable, and depends on the complex relationships between the host, the bacteria and the drug. As far for what concerns the host, differences in the pharmacokinetic profiles and in pharmacodynamics, in terms also of predisposition to adverse effects, can severely impact on the response to therapy, as well as on drug induced toxicity. Pharmacokinetic differences concern absorption, distribution, metabolism and renal excretion, and interindividual differences in genes encoding phase I and phase II hepatic enzymes and transporters are of particular interest [3,4]. In addition, adverse drug reactions, and in particular, immunologically mediated, type B idiosyncratic reactions, have been associated with variations in major histocompatibility complex (MHC) molecules of the host, and increasing evidence suggests that specific human leukocyte antigen (HLA) alleles can predispose one to hypersensitivity reactions to various antibiotics [5].

This review will consider the most relevant genetic variations that can result in altered response or unexpected toxicity of antimicrobial agents employed in clinical practice.

## 2. Antibiotics

### 2.1. The Beta-Lactams

#### 2.1.1. Penicillins

Penicillin G, the progenitor of all beta-lactams, was introduced in the early 1940s; despite the widespread occurrence of bacterial resistance, penicillins continue to be employed as first choice agents in a number of bacterial infections. Due to their mechanism of action, i.e., the inhibition of the last step in the synthesis of peptidoglycan, a component of the bacterial cell wall not present in eukaryotes, penicillins and the other beta-lactams are extremely safe, and the main problem during therapy is the occurrence of a wide spectrum of hypersensitivity reactions, including skin allergy and anaphylaxis [6].

##### The Aminopenicillins, Ampicillin and Amoxicillin

Among beta-lactams the aminopenicillins, and in particular amoxicillin, are commonly employed, in particular for the therapy of upper respiratory infections. In patients in therapy with these agents, skin rashes are common, with a frequency of 9.1%, and are particularly frequent in subjects with concomitant viral infections such as mononucleosis [7].

##### Amoxicillin Clavulanate

**Figure 1:**
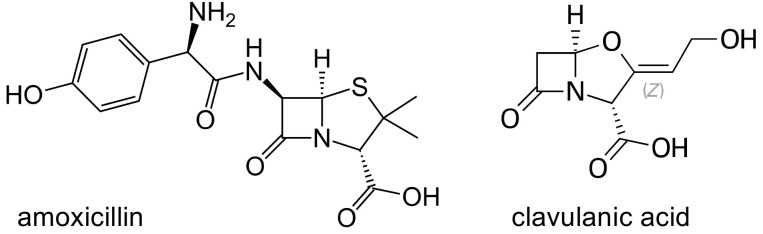
Chemical structures of amoxicillin and clavulanic acid.

Being susceptible to the action of beta-lactamases, amoxicillin is frequently combined with the beta-lactamase inhibitor clavulanic acid (Figure 1), and this association represents one of the most commonly prescribed antibacterial agents worldwide. Clavulanic acid is a suicide inhibitor, that binds beta- lactamases produced by Gram-positive and Gram-negative bacteria, preventing the inactivation of the penicillin. The combination is usually very well tolerated, however; an idiosyncratic liver toxicity has been described [8,9,10]. In a population study conducted in Iceland [11], the incidence of drug induced liver injury (DILI) was 19.1 per 100,000 persons per year; antibiotics, and among them amoxicillin clavulanate, have been identified as the leading cause of this side effect. Indeed, around 17% of all severe cases of drug induced liver disease leading to hospitalization were caused by this antibiotic combination [8,12], most probably by the clavulanic acid component [13]. The toxicity is usually benign, and most patients completely recover without long term sequelae after therapy interruption [9,14], however, damage can rarely proceed to acute liver failure, leading to death or liver transplant [15]. Although the mechanism of this side effect is still unclear, an immunologic reaction, linked to drug hapten presentation via the MHC, has been hypothesized. Studies have described an association between drug induced liver injury and the HLA class II allele DRB1*15:01, with odds ratios (OR) between 2.6 and 10 [16,17,18]. The results were not confirmed by a Spanish study [19] on 27 cases; in this cohort of patients, a significantly higher frequency of DQB1*06:14 and no association with DRB1*15:01 were demonstrated; however, these differences were ascribed to different genotyping techniques, differences in the studied populations, or to a higher percentage of cases with hepatocellular damage in comparison to cholestatic/mixed cases in the Spanish study.

**Figure 2:**
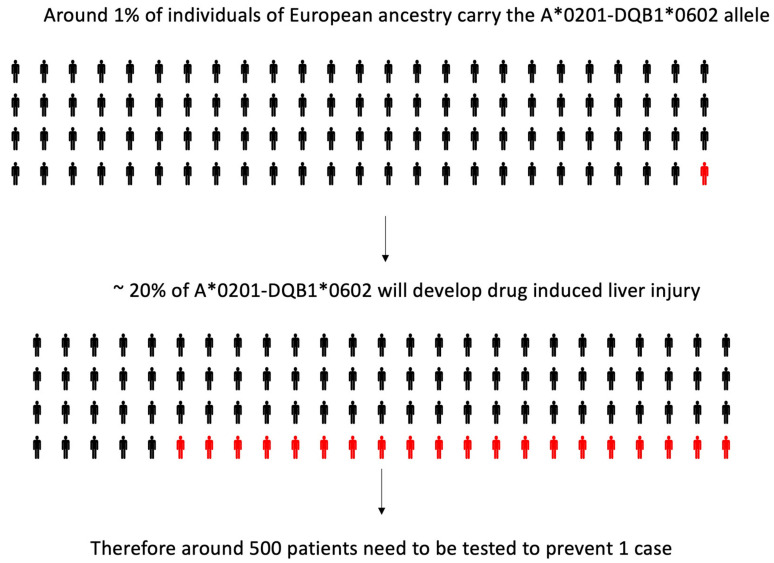
Limited positive predictive value of human leukocyte antigen (HLA) risk alleles for amoxicillin-clavulanate-induced liver injury: on the basis of data published by Lucena and colleagues [20].

In 2011 [20], a genome-wide association (GWA) study was performed, in which 822,927 single nucleotide polymorphisms (SNP) were tested on 201 White European and US subjects who had developed amoxicillin clavulanate induced liver injury and 532 genetically matched controls. An association was found with many loci in the MHC: the strongest effect was evident with an HLA class II variant (rs9274407, *p* = 4.8 × 10^−14^), which correlated with rs3135388, a tag SNP of HLA-DRB1*15:01-DQB1*06:02, previously associated with amoxicillin clavulanate induced liver injury [8,16,17,18]. HLA-A*02:01 (rs2523822) was also independently associated with DILI (*p* = 1.8 × 10^−10^). In addition, a statistical interaction (*p* = 0.0015) was shown, with the most significant class I and II SNPs, showing that when both minor alleles were present, the risk was larger than expected, based on the effect of each variant in the univariate analysis. The association of HLA-A*02:01 (*p* = 2 × 10^−6^) and HLA-DQB1*06:02 (*p* = 5 × 10^−10^) and their interaction (*p* = 0.005) was confirmed by high-resolution HLA genotyping on 177 cases and 219 controls. However, the HLA genotypes have low positive predictive values, and hence a limited utility as predictive or diagnostic biomarkers [20] (Figure 2).

In a subsequent study by the same authors, high resolution genotyping of the HLA loci A, B, C, DRB1 and DQB1 was performed in 75 amoxicillin clavulanate DILI cases and 885 controls. Class I alleles A*30:02 (*p/pcorrected* = 2.6 × 10^−6^/5 × 10^−5^, OR = 6.7) and B*18:01 (*p/pc* = 0.008/0.22, OR = 2.9) were more frequent in subjects who presented liver toxicity than in controls. In cholestatic/mixed cases, the class II allele combination DRB1*15:01-DQB1*06:02 (*p/pc* = 5 × 10^−4^/0.014, OR = 3.0) was more frequent. Age was lower and hospitalization was more frequent in A*30:02 and/or B*18:01 carriers (54 vs. 65 years, *p* = 0.019) than in the DRB1*15:01-DQB1*06:02 carriers. B*18:01 (*p/pc* = 0.015/0.42, OR = 5.2) and DRB1*03:01-DQB1*02:01 (*p/pc* = 0.0026/0.07, OR = 15) frequencies were higher in cases with delayed onset, compared to patients without delayed onset, while the opposite was seen with DRB1*13:02-DQB1*06:04 (*p/pc* = 0.005/0.13, OR = 0.07). The authors concluded that HLA class I and II alleles influence the phenotypic expression, latency presentation and severity of amoxicillin clavulanate DILI in Spanish patients [21].

Finally, another genome-wide association study was conducted on a multiethnic cohort of 2048 patients, with DILI and 12,429 controls of European (1806 cases and 10,397 controls), African American (133 cases and 1314 controls), and Hispanic (109 cases and 718 controls) ancestry. A validation cohort of 113 Icelandic cases and 239,304 controls was also studied. Idiosyncratic DILI was associated with rs2476601, a nonsynonymous polymorphism that encodes a tryptophan to arginine substitution in the protein tyrosine phosphatase, non-receptor type 22 gene (*PTPN22*) (OR = 1.44; 95% CI, 1.28–1.62; *p* = 1.2 × 10^−9^); these data were replicated in the validation cohort (OR = 1.48; 95% CI, 1.09–1.99; *p* = 0.01). The variant was associated with the risk of liver injury caused by various drugs (OR = 1.37; 95% CI, 1.21–1.56; *p*= 1.5 × 10^−6^; allele frequency = 11.5%), but in particular, by amoxicillin clavulanate in persons of European ancestry (OR = 1.62; 95% CI, 1.32–1.98; *p* = 4.0 × 10^−6^; allele frequency = 13.3%); indeed, rs2476601 doubled DILI risk in subjects with the HLA A*02:01 and DRB1*15:01 risk alleles. Of interest, the *PTPN22* variant has been previously associated with an increased risk of autoimmune diseases, further supporting the hypothesis that alterations in immune regulation contribute to idiosyncratic DILI [22]

##### Flucloxacillin

**Figure 3:**
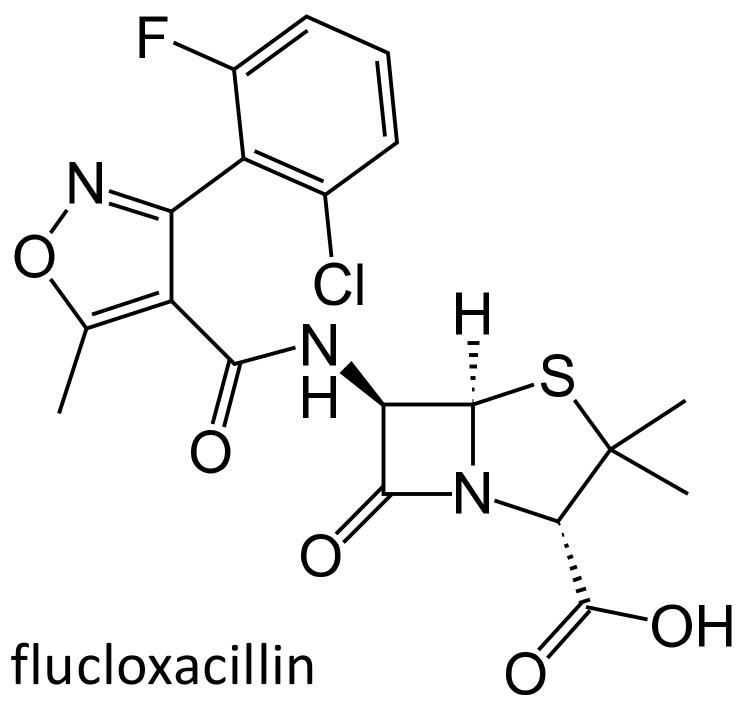
Chemical structure of flucloxacillin.

One major problem of penicillins is their susceptibility to inactivation by bacterial produced beta-lactamases; the penicillinase-resistant penicillins are a class of antibiotics that are not hydrolyzed by the staphylococcal enzymes. Unfortunately, an increasing number of *Staphylococcus aureus* and *Staphylococcus epidermidis*, known as the methicillin resistant *Staphylococcus aureus* (MRSA) or methicillin resistant *Staphylococcus epidermidis* (MRSE) strains, have become resistant also to these agents, through the expression of penicillin binding proteins with low affinity [23]. Clinically employed penicillinase-resistant penicillins belong to the isoxazolyl penicillin class, which includes oxacillin, cloxacillin, dicloxacillin and flucloxacillin (Figure 3).

For flucloxacillin, a rare, but potentially serious complication is a cholestatic hepatitis, which affects 1–2 per 1000 individuals treated with the drug, whose pathogenesis is not clear. A GWA study using 866,399 markers was conducted in 51 patients with flucloxacillin DILI, and 282 sex and ancestry matched controls. An association peak in the MHC region on chromosome 6 was observed, and the strongest association (*p* = 8.7 × 10^−33^) was seen for rs2395029; in subjects of European origin, this marker is in complete linkage disequilibrium with HLA-B*57:01. Cases and drug-exposed controls were further genotyped, confirming that HLA-B*57:01 allele (rs2395029) carriers have an 80 fold increased risk of DILI when treated with flucloxacillin, as compared to patients with no HLA-B*57:01 alleles. The association was also replicated in another cohort of 23 cases. In addition, in HLA-B*57:01 carriers, rs10937275, an intronic SNP in beta-galactoside alpha-2,6-sialyltransferase 1 (*ST6GAL1*) on chromosome 3, also showed genome-wide significance (OR = 4.1, *p* = 1.4 × 10^−8^) [24]. *ST6GAL1* encodes an enzyme that catalyzes the transfer of sialic acid, and an increased expression of this enzyme has been demonstrated in acute inflammation in the rat [25].

To confirm these findings, the same authors performed a GWA study on 197 patients with flucloxacillin-induced DILI and 6835 controls. The major risk factor was HLA -B*57:01 (OR = 36.62; *p* = 2.67 × 10^−97^), and an association was seen also with HLA-B*57:03 (OR = 79.21; *p* = 1.2 × 10^−6^). Within the HLA-B protein sequence, imputation showed that valine in position 97 in the HLA-B, common to HLA-B*57:01 and HLA-B*57:03, had the stronger association (OR: 38.1; *p* = 9.7 × 10^−97^) in comparison to other residues, such as the more common arginine and serine, that had, on the other hand, a protective effect. HLA-B*57 was not associated with DILI induced by other isoxazolyl penicillins (*n* = 6) or amoxicillin (*n* = 15). In addition, penicillin-related liver damage was not significantly associated with non-HLA [26]. As this complication is, however, rare, the positive prediction of tests on the HLA-B*57:01 allele is very low (0.12%), and therefore routine testing seems, at present, impractical. A regular monitoring of the patient’s liver function is mandatory and an alternative antibiotic should be prescribed if liver enzymes and/or bilirubin levels become elevated.

Studies have suggested that flucloxacillin is an agonist of the human pregnane X receptor (PXR). To evaluate the role of PXR in flucloxacillin toxicity, human hepatocytes were treated for 72 h with 500 µM flucloxacillin, and an expression microarray analysis was performed. Significant changes in expression were evident for seventy-two probe sets representing 50 different genes; most genes with greater than 3-fold changes were known to be responsive to rifampicin, suggesting a regulation mediated by the PXR. Reporter gene experiments confirmed that flucloxacillin is a PXR agonist. Three SNPs in the *PXR* gene were evaluated in 51 patients with flucloxacillin DILI, 64 controls treated with the drug without developing toxicity, and 90 population controls, all of white European origin. The C-25385T *PXR* polymorphism (rs3814055) showed a different distribution between flucloxacillin DILI cases and drug treated controls, and an increased risk of the adverse event was associated with the CC genotype (OR: 3.37, 95% CI: 1.55–7.30, *p* = 0.0023). Interestingly, luciferase reporter assay demonstrated that the C allele had a lower promoter activity in comparison with the T allele. The authors concluded that flucloxacillin is a PXR agonist at therapeutic concentrations and the C-25385T polymorphism in the *PXR* gene is a risk factor for flucloxacillin-induced DILI [27].

#### 2.1.2. Cephalosporins

**Figure 4:**
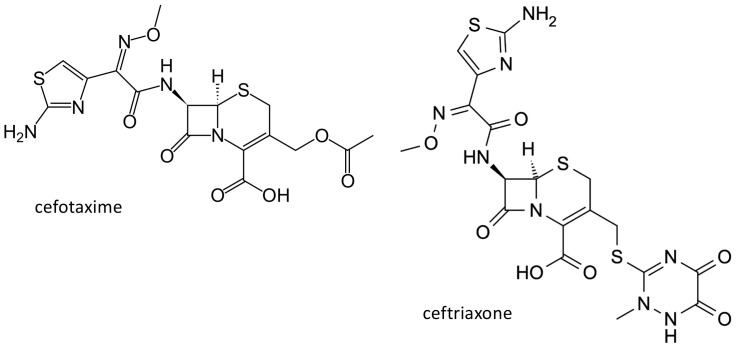
Chemical structure of the third generation cephalosporins cefotaxime and ceftriaxone.

Cephalosporins belong to the beta-lactam antibiotics, and have been obtained from the cultures of *Cephalosporium acremonium*, isolated in 1948 by the Italian pharmacologist Giuseppe Brotzu from the sea in the proximity of a sever outlet near Cagliari in Sardinia. Cefotaxime (Figure 4) is a third-generation cephalosporin, and is largely employed in the treatment of serious Gram-negative infections. The drug is excreted mainly by the renal route, through active tubular secretion [28]. The organic anion transporter (OAT) 1, 3 and 4, expressed on the basolateral membrane of the proximal tubular cells, play an important role in the renal excretion of organic anions, including many cephalosporins [29,30,31]. In addition, cefotaxime inhibits estrone sulphate uptake by OAT3 overexpressing cells [32]. Genetic polymorphisms of the OAT3 gene (*SLC22A8*) have been described; in the 1000 genome project, a nonsynonymous Ile305Phe variant (rs11568482) was reported, exclusively in subjects of Asian ancestry, with an allele frequency of 3.6–6% [33]. In HEK293-Flp-In cells, the maximal cefotaxime transport activity, Vmax, was lower in cells with the variant in comparison with the wild type OAT3 (159 ± 3 nmol×(mg protein)(−1)/min (mean ± SD) versus 305 ± 28 nmol×mg protein)(−1)/min, (mean ± SD), *p* < 0.01), while no difference was observed for the Michaelis–Menten constant values (Km) [34]. Cefotaxime renal clearance was significantly lower in healthy volunteers heterozygous for the Ile305Phe variant (84.8 ± 32.1 mL/min, mean ± SD, *n* = 5), in comparison with subjects homozygous for the reference allele (158 ± 44.1 mL/min, *n* = 10; *p* = 0.006). Moreover, the net secretory component of cefotaxime renal clearance was reduced in heterozygous volunteers (33.3 ± 31.8 mL/min (mean ± SD)) compared with wild type homozygotes (97.0 ± 42.2 mL/min (mean ± SD), *p* = 0.01) [35]. However, it is not clear if this polymorphism could be responsible for the variability of drug levels observed in patients treated with this drug.

Another largely used third generation cephalosporin is ceftriaxone (Figure 4). This agent is transported by the multidrug resistant-associated protein (MRP)2, encoded by the ATP binding cassette, subfamily C, member 2 (*ABCC2*) gene, and by the breast cancer resistance protein, encoded by the *ABCG2* gene [36]. These proteins, like P-glycoprotein (encoded by the *ABCB1* gene), belong to the ATP-binding cassette transporters superfamily, and are expressed in various tissues among which the blood brain barrier, where they exert an important role in the protection of the central nervous system from drugs and xenobiotics [37,38]. Furthermore, solute carrier organic anion transporting protein (OATP) transporters are expressed on the blood brain barrier and probably have a similar role in controlling drug disposition into the central nervous system [39,40]. Among OATPs, OATP1A2, encoded by the solute carrier organic anion transporter family member *(SLCO)1A2* gene, in particular, is involved in the penetration of various drugs into the brain [39,40]. Ceftriaxone concentrations were measured by high-performance liquid chromatographic methods in plasma and cerebrospinal fluid of 43 adult patients with central nervous system infections, and were related to biometric, demographic, genetic (*ABCB1*, *ABCC2*, *ABCB11*, *ABCG2*, and *SLCO1A2* polymorphisms) and pathological features. *ABCC2* 1249 rs2273697 (*p* = 0.027) and *ABCG2* 1194 + 928 rs13120400 (*p* = 0.015) variants were significantly associated with ceftriaxone concentrations in cerebrospinal fluid and with cerebrospinal fluid to plasma ratios [41].

#### 2.1.3. Beta-Lactam Induced Neutropenia

As already mentioned, all beta lactams are extremely safe drugs, however, in around 5–15% of patients treated for periods of more than 10 days with high doses of these antibiotics, neutropenia develops [42]. While this complication is completely reversible when therapy is interrupted, an increased frequency of infections and other potentially severe complications can occur [43]. The molecular mechanisms of neutropenia are still unclear, however, Hahn and collaborators have shown that patients with a homozygous 3348 A > G coding synonymous polymorphism in *MRP4* (rs1751034) were more likely to develop this side effect, although the difference was not significant, probably for the limited sample size [44]. Beta-lactams are substrates of MRP4 expressed on renal proximal tubule cells, on CD34+ stem cells and other bone marrow cells [45]; in addition, increased renal and plasma concentrations of beta lactams have been demonstrated in mouse models lacking MRP4 and OAT3 [46,47]. The authors suggest that this polymorphism should be further investigated in a larger cohort of subjects.

### 2.2. The Macrolides

**Figure 5:**
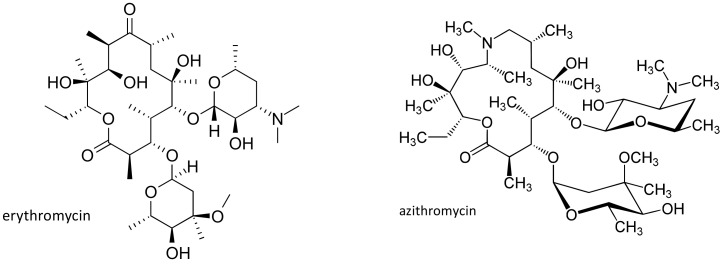
Chemical structures of erythromycin and azithromycin.

Another widely used antibiotic class is represented by macrolides, that are employed in the treatment of different infections, in particular those involving the respiratory tract. Erythromycin (Figure 5), the product of a strain of *Streptomyces erythreus*, was discovered in 1952 by McGuire and coworkers [48], and has been the first agent of this class to be introduced in the clinics [49,50]. Many other semisynthetic derivatives have been subsequently obtained, among which the most used are azithromycin (Figure 5) and clarithromycin, that, at least in Western countries, have almost completely replaced erythromycin [51].

#### 2.2.1. Erythromycin

Erythromycin undergoes extensive hepatic metabolism by cytochrome P450 (CYP) 3A4, and is also a substrate for the drug transporter MRP2, encoded by the *ABCC2* gene, and for its murine ortholog, *Abcc2*; these transporters are highly expressed on the biliary surface of hepatocytes. In 2011, Franke and coworkers [52] observed that, in *Abcc2* knockout mice, the deficiency of the protein was associated with a significant increase in erythromycin metabolism, with no change in Cyp3a protein expression and activity. In addition, in a cohort of 108 cancer patients, homozygosity for the -24 C > T variant in the *ABCC2* promoter (rs717620), with reduced transport function, was correlated with an increased metabolism of erythromycin (*p* = 0.013), but not with midazolam clearance. These data suggest that erythromycin metabolism can be modified by a reduced MRP2 function, through an alteration of its biliary secretion, and subsequent increased permanence of the drug in the hepatocyte, therefore independently of changes in CYP3A4 activity [52].

Erythromycin is also a substrate for OATP1B1, encoded by the *SLCO1B1* gene, expressed on the basolateral surface of hepatocytes. The transport of erythromycin was reduced by ~50% in stably transfected Flp-In T-Rex293 cells expressing OATP1B1*5 (V174A), in comparison to OATP1B1*1A (wild type). A 52% decrease in the metabolic rate of erythromycin (*p* = 0.000043) was observed in mice deficient of the Oatp1b2 transporter, while in 91 patients with cancer undergoing erythromycin breath test, the c.521T > C variant in *SLCO1B1* (rs4149056), encoding OATP1B1*5, was associated with a reduction in erythromycin metabolism (*p* = 0.0072) [53].

#### 2.2.2. Azithromycin

Due to structural differences from erythromycin and clarithromycin, azithromycin does not interact with CYP3A4, however, this drug is the substrate of polymorphic transporters. In rats, Sugie et al. [54] have shown that azithromycin is a substrate for P-glycoprotein and MRP2; these transporters contribute to the biliary and intestinal excretion of the antibiotic. 

The effect of *ABCB1* polymorphisms on the pharmacokinetics of azithromycin was then evaluated in 20 Chinese Han healthy volunteers (6 with 2677GG/3435CC, 8 with 2677GT/3435CT, 6 with 2677TT/3435TT). After a single oral dose of 500 mg azithromycin, subjects with the 2677TT/3435TT genotype had a significantly lower C_max_ (468.0 ± 173.4 ng × h/mL), in comparison to those with 2677GG/3435CC (911.2 ± 396.4 ng × h/mL, *p* = 0.013). On the contrary, the t_max_ value was higher in subjects with 2677TT/3435TT (2.0 ± 0.5 h), in comparison with those with the 2677GG/3435CC (1.4 ± 0.4 h) genotypes (*p* = 0.026) [55].

### 2.3. The Aminoglycosides

**Figure 6:**
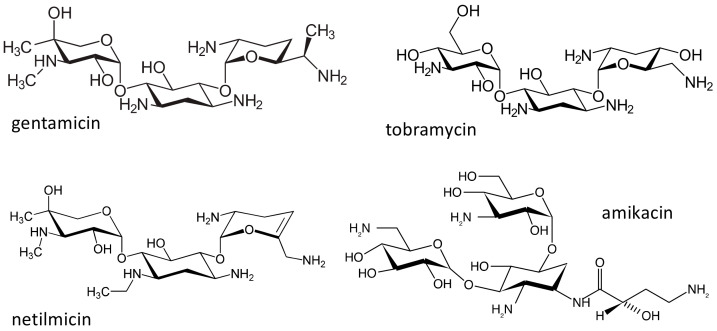
Chemical structure of the aminoglycosides gentamicin, tobramycin, netilmicin and amikacin.

The aminoglycosides (Figure 6) are a class of natural products or semisynthetic derivatives of compounds produced by soil actinomycetes. Streptomycin was the first agent of this class to be isolated in 1943 from a strain of *Streptomyces griseus*, and was subsequently followed by many other compounds, among which, gentamicin, tobramycin, netilmicin and amikacin are the most used. These antibiotics are particularly effective against Gram-negative bacteria and, despite their toxicity, continue to be employed in the clinics, as they retain a good activity against multidrug resistant pathogens [56]. The main toxicities of aminoglycosides are a reversible nephrotoxicity and an irreversible bilateral ototoxicity; more common with high doses and prolonged treatments [57,58]. Aminoglycosides exert their antibacterial activity by binding to the 16S ribosomal RNA (rRNA) in the 30S subunit of the bacterial ribosome, interfering with protein synthesis. Bacterial ribosomes have many similarities with mammalian mitochondrial ribosomes; therefore the small subunit of mitochondrial ribosomes could be a target site for aminoglycosides. Authors have suggested that mutations in mitochondrial DNA, in particular in the 12S rRNA genes, could be related to an increased sensitivity and toxicity to these antibiotics. Several 12S rRNA mutations have been identified [59]: in three Chinese families affected by ototoxicity induced by aminoglycosides, and in an Arab-Israeli family with non-syndromic maternally inherited deafness, an m1555A > G transition has been described [60]. In subsequent studies, the frequency of this mutation in subjects with aminoglycoside ototoxicity varied from 5% to 33% in the examined cohorts [59], and patients with the 1555G allele had a greater risk of experiencing aminoglycoside-induced hearing loss, as compared to patients with the 1555A allele, even if other genetic and clinical factors may also influence the risk of aminoglycoside-induced hearing loss. After exposure to aminoglycosides, penetrance of deafness in subjects with the m1554A > G mutation is close to 100% [61]. The variant has been genotyped in the Avon Longitudinal Study of Parents and Children (ALSPAC) cohort, and a population prevalence of 0.19% (95% CI 0.10–0.28) was found [62]. This population prevalence was confirmed in the Blue Mountains Hearing Study. In this cohort, of 2856 subjects over the age of 49 years, 6 had homoplastic m1554A > G mutations, with a prevalence of 0.21% (95% CI 0.08–0.46) [63].

Other mutations in 12S rRNA were also identified, in particular an m1494 C > T transition in a large Chinese family with aminoglycoside-induced and non-syndromic deafness [64], but the incidence of this mutation, maternally transmitted, was much lower [59]. Other variants have been also associated with aminoglycoside ototoxicity, but all were extremely rare [59]. It has been suggested that the 1555A > G and 1494C > T mutations create a new C-G or A-U base pair, making the human mitochondrial ribosome more similar to the bacterial one, and modifying the binding site for aminoglycosides. As a consequence, in subjects carrying these polymorphisms, an impairment of protein synthesis and defects in cell respiration occurs, that can be the con cause of hearing loss after exposure to these antibiotics in patients carrying these mutations [59]. 

In the clinics, these predisposing mutations are not routinely screened, however, for subjects that are predicted to be treated for prolonged periods with this class of antibiotics, such as patients with multidrug resistant tuberculosis or children with acute lymphoblastic leukemia, screening for 12S rRNA mutations, when balanced against the cost of lifelong deafness or need for cochlear implantation, is cost-effective and should be proposed; in the presence of such mutations, the use of aminoglycosides should therefore probably be avoided [59].

### 2.4. The Fluoroquinolones

**Figure 7:**
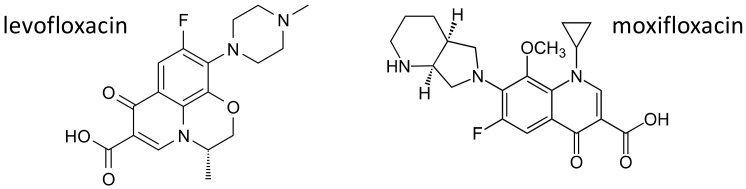
Chemical structures of the fluoroquinolones levofloxacin and moxifloxacin.

For their broad spectrum and excellent pharmacokinetics, fluoroquinolones have been largely used for common infective conditions. Some serious toxicities have been, however, described over the years, and alerts from regulatory agencies now limit the use of these drugs [65,66]. Among fluoroquinolone induced adverse effects, central nervous system alterations, particularly in elderly patients, have been described [67]. As already mentioned, ABC transporters play an important role in limiting the penetration of drugs and xenobiotics through the blood brain barrier [68], and fluoroquinolones are substrates of multiple ABC transporters [69,70,71,72]. A case study [73] describes a 45-year-old patient with no known predisposing conditions, who developed two episodes of generalized seizures after treatment with levofloxacin (Figure 7). Polymorphisms of the efflux transporters genes *ABCB1*, coding for P-glycoprotein, and *ABCG2*, coding for BCRP were evaluated. The patient carried one variant allele for the most studied SNPs in *ABCB1* (1236C > T, 2677G > T/A and 3435C > T) [74], and a functional polymorphism (421C > A) in *ABCG2* gene; on the contrary, he was wild type for two polymorphisms (38T > C and 516A > C) in *SLCO1A2*, coding for the organic anion transporter OATP1A1, also involved in levofloxacin transport [75,76]. The authors suggest that, in this patient, neurological side effects could be related to a genetically determined reduced activity of P-glycoprotein and BCRP, with increased penetration of levofloxacin through the blood brain barrier [73].

Among fluoroquinolones, moxifloxacin (Figure 7), besides being effective against a wide range of Gram-positive and Gram-negative bacteria, including anaerobes [77], also exhibits bactericidal activity against mycobacterium tuberculosis, and is now included in several regimens for the treatment of drug-susceptible and drug-resistant tuberculosis [78], or in patients who develop side effects to first line antituberculosis agents [79]. Moxifloxacin exhibits significant pharmacokinetic variability, in particular in patients with tuberculosis, [80], and variations in genes encoding for drug metabolizing enzymes and drug transporters have been suggested as possible causes of this variability.

Naidoo and collaborators [79] evaluated the effect of variability in uridine 5′diphosphate (UDP) glucuronosyltransferase *(UGT)1A* and *ABCB1* genotypes on moxifloxacin pharmacokinetics. in a population of 172 South Africa tuberculosis patients, however, pharmacokinetic data were available only for 58 subjects. Moxifloxacin is metabolized by sulphotransferase and UGTs. These are a family of highly polymorphic cytosolic enzymes, and polymorphisms have been demonstrated to be responsible for variations in the pharmacokinetics of a number of drugs [81,82]. UGT1A1 is the main isoform involved in moxifloxacin metabolism: the rs8175347 *UGT1A* SNP was significantly associated with a lower clearance of the drug, while rs3755319 was associated with a higher clearance. In addition, the *ABCB1* SNP rs2032582 was associated with a reduced bioavailability of the drug [83]. Indeed, moxifloxacin is also a substrate for the multidrug transporter P-glycoprotein encoded by the *ABCB1* gene, and the 3435CC variant (rs1045642) was shown to affect moxifloxacin absorption in a small study conducted in healthy subjects; the association, however, was not statistically significant in multivariate analysis when adjusted for weight [84]. 

A second study [85] evaluated the effect of the –11187G > A variant in the *SLCO1B1* gene (rs4149015) in 49 participants, in a Tuberculosis Trials Consortium pharmacokinetic study. The authors found that the moxifloxacin area under the concentration-time curve from 0 to 24 h (AUC_0–24_) and the Cmax were significantly higher in patients with the variant; in particular, the median AUC_0–24_ was 46% higher (*p* = 0.005) and the Cmax was 30% higher (*p* = 0.009) in 4 subjects (8%) with the *SLCO1B1* –1187 AG genotype, in comparison to the 45 patients with the wild type GG genotype. The authors suggest that this increase in blood drug levels could be important for one of moxifloxacin side effects; indeed, the fluoroquinolone induces a concentration dependent prolongation of the QTc interval [86], associated with cardiac arrhythmias in particular, in patients with known pro-arrhythmic conditions [33,34].

### 2.5. Vancomycin

**Figure 8:**
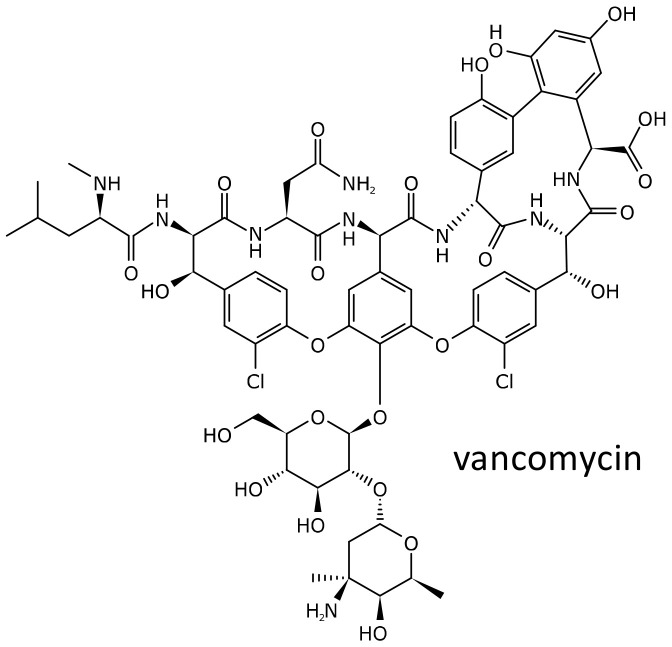
Chemical structure of vancomycin.

Vancomycin (Figure 8) is the originator of the glycopeptide class of antibiotics. Discovered in the 1950s, the antibiotic soon became available in the clinics, but, due to its toxicity, was quickly discarded in favor of other, safer and more effective compounds [87]. However, the diffusion of infections caused by methicillin resistant *Staphylococcus aureus* and the rise of *Clostridium difficile* enterocolitis led to the resurgence in its use [87]. Several side effects have been described, among which are the red neck or red man syndrome, due to a direct histamine releasing effect on mast cells, and nephro and ototoxicity. In addition, vancomycin is an important cause of the severe hypersensitivity syndrome drug reaction with eosinophilia and systemic symptoms (DRESS). Patients who had presented a probable vancomycin-induced DRESS were matched 1:2 with subjects treated with the antibiotic without exhibiting the side effect, based on sex, race, and age, by using a deidentified electronic health record database. Associations between DRESS and carriage of HLA class I and II alleles were assessed by means of conditional logistic regression. Among 23 subjects of predominantly European ancestry with vancomycin-associated DRESS, 19 (82.6%) carried HLA-A*32:01, in comparison to 0 (0%) of 46 matched vancomycin tolerant controls (*p* = 1 × 10^−8^) and 6.3% of the BioVU cohort (*n* = 54,249, *p* = 2 × 10^−16^). The strong association observed suggests that HLA-A*32:01 testing could be proposed to improve the safety of vancomycin [88].

As already discussed, vancomycin can also be nephrotoxic; age, creatinine clearance, vancomycin dose and dosing interval, and concurrent nephrotoxic drugs are risk factors, but are not able to always predict this side effect. To identify potential genomic risk factors, a GWA study was performed in 489 European American patients treated with vancomycin, and findings were subsequently validated in three independent cohorts, for a total of 439 European American subjects. In the primary cohort, the most significant SNP correlated with increased serum creatinine levels was rs2789047, at chromosome 6q22.31 locus (risk allele A, beta = −0.06, *p* = 1.1 × 10^−7^). SNPs in this region had consistent directions of effect in the validation cohorts (meta-*p* = 1.1 × 10^−7^). This region on chromosome 6 includes the *TBC1D32/C6orf170* (encoding a ciliary protein) and *GJA1* (encoding connexin 43) genes, and variations may affect the risk of vancomycin-induced kidney injury. The rs2789047 variant was, however, not associated with vancomycin through concentration or elimination rate constant (ke), suggesting a mechanism not related to the drug pharmacokinetics [89].

### 2.6. Daptomycin 

**Figure 9:**
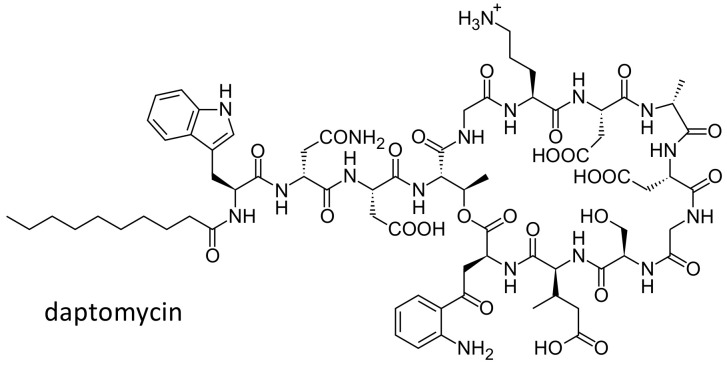
Chemical structure of daptomycin.

Daptomycin (Figure 9) is a lipopeptide antibiotic effective against Gram-positive bacteria and approved for the treatment of complicated skin and soft tissues infections caused by Gram positive-cocci, *Staphylococcus aureus* bacteremia and right sided *Staphylococcus aureus* endocarditis. In THP-1 macrophages and MDCK cells, the drug was shown to be a substrate of P glycoprotein [90,91]. In 23 daptomycin treated Caucasian patients, three SNPs, in the *ABCB1* gene 3435C > T (rs1045642), 1236C > T (rs1128503) and 2677G > T (rs2032582), were studied. The median dose-normalized AUC_0-24_ was higher in patients with the homozygous variant TT genotype, as compared to those with the CT or CC genotype. Indeed, patients with the TT genotype had a decreased clearance of daptomycin, resulting in increased concentrations of the drug. Although the study was conducted in a very small cohort of patients, the results suggest that this polymorphism could explain the high variability in pharmacokinetics observed in the clinics [92].

### 2.7. Linezolid

**Figure 10:**
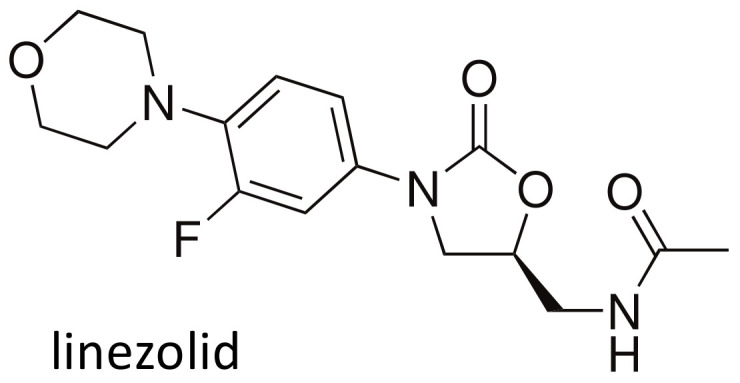
Chemical structure of linezolid.

Linezolid (Figure 10) is the originator of a relatively new class of protein synthesis inhibitors, the oxazolidinones, effective against the majority of Gram-positive bacteria, including multidrug resistant pathogens [93]. The compound is also employed for the treatment of multidrug resistant tuberculosis [78,94]. Linezolid pharmacokinetics is often unpredictable, in particular in critically ill patients [95,96]; it has been suggested that this variability could be linked to the altered expression of transmembrane transporters. In 2018, Allegra and collaborators [97] evaluated the most common polymorphisms in many drug transporters in 27 critically ill patients treated with linezolid. A significant effect of the 3435C > T polymorphism (rs1045642) in the *ABCB1* gene, coding for P-glycoprotein, on linezolid clearance was found, with values of 13.19 ± 6.81 l/h (mean ± SD) in wild type 3435CC homozygotes and 7.82 ± 4.21 l/h for 3435CT/TT subjects (*p* = 0.042). A difference was found between wild type and all other patients, also for volume of distribution (37.43 ± 7.20 L vs. 46.72 ± 14.67 L, *p* = 0.038) and terminal half-life (2.78 ± 2.56 h vs. 5.45 ± 3.94 h, *p* = 0.044), while only a trend was observed for AUC_0-24_ (130.85 ± 121.07 h·mg/L vs. 208.59 ± 117.85 h·mg/L, *p* = 0.130). The authors [97] suggest that polymorphisms in the *ABCB1* gene could influence linezolid pharmacokinetics and hypothesize the incorporation of this pharmacogenetic assay into the clinics to personalize treatment. 

### 2.8. Minocycline

**Figure 11:**
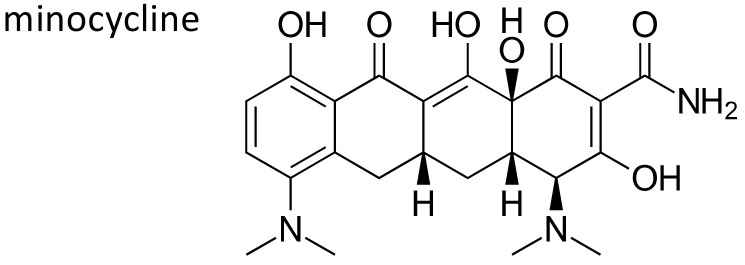
Chemical structure of minocycline.

Minocycline (Figure 11) is a member of tetracyclines, bacteriostatic antibiotics with a wide spectrum of activity, employed as first line therapy in infections caused by rickettsiae, mycoplasma and chlamidiae, and in respiratory, skin and soft tissue infections caused by MRSA. As already mentioned, antibiotics are the leading cause of drug induced liver toxicity [10], and among them, minocycline has been also implicated as a cause of this side effect, often associated to other characteristic clinical features, including systemic arthralgias and production of autoantibodies [98]. The side effect, rare but potentially severe, is usually seen after prolonged use of the drug for acne. Twenty five Caucasian patients who presented DILI underwent GWA genotyping and were compared to unexposed ancestry matched controls. A significant association was observed between HLA-B*35:02 allele and risk for liver toxicity (16% vs. 0.6%; OR: 29.6, 95% CI: 7.8–89.8, *p* = 2.5 × 10^−8^). The association was confirmed by sequence-based HLA typing. The HLA-B*35:02 allele could represent a useful diagnostic marker of minocycline induced liver injury, and testing could reduce the risk of this side effect [99]. 

### 2.9. Clindamycin

**Figure 12:**
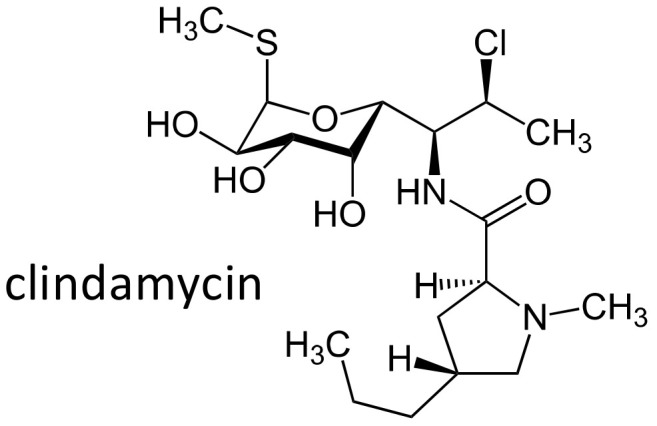
Chemical structure of clindamycin.

Clindamycin (Figure 12) is a lincosamide with a wide spectrum that includes aerobic and anaerobic bacteria. In addition, the compound inhibits the synthesis of toxic shock syndrome toxins, through the inhibition of protein synthesis.

The most fearful side effect of the drug is severe *Clostridium difficile* enterocolitis, however, cutaneous adverse drug reactions have also been reported. To assess whether HLA alleles are associated with clindamycin-related cutaneous adverse drug reactions in the Han Chinese population, an association study of 12 subjects with the side effect, 26 clindamycin-tolerant subjects and 279 controls was performed [100]. Six out of 12 clindamycin-induced cutaneous reaction patients carried HLA-B*51:01, and the frequency was higher in comparison to controls with the control group (*p* = 0.0006; OR = 9.731, 95% CI: 2.927–32.353) and to clindamycin-tolerant subjects (OR = 24.000, 95% CI: 3.247–177.405), suggesting that HLA-B*51:01 is a risk allele for clindamycin-related cutaneous adverse reactions in Han Chinese [100].

## 3. Conclusions

Pharmacogenetic biomarkers are limited for antibiotics, probably because these molecules are often able to interact specifically with microbial targets, with limited interaction with human ones. Moreover, many antibiotics are highly hydrophilic, and therefore are not substrate of the major hepatic biotransformation pathways. However, variants in some drug transporters, involved in the cellular transport of antibiotics, can be associated with interindividual variability in the clearance and effects of these drugs (Table 1). Limited clinical impact of variants associated with antibiotic effects could be related also to the relatively short-term therapies performed, with many agents that are administered to patients generally for a limited number of days. Interestingly, some of the pharmacogenetic studies mentioned in this review involve antibiotics used also to treat tuberculosis, that, on the contrary, requires long treatments. On these bases, many of the pharmacogenetic traits of interest are related to immune-mediated reactions, therefore involving HLA alleles at the molecular level (Table 2). These markers, while often highly significant and reproducible, generally involve rare alleles, and therefore the clinical utility of the pharmacogenetic biomarkers is limited by a low positive predictive value. Indeed, so far, in terms of actionable pharmacogenetic markers, established guidelines, such as those reported on the PharmGKB website, that lists dosing guidelines from CPIC, DPWG, CPDNS and other authoritative societies, report only one guideline for flucloxacillin and HLA-B alleles. Interestingly, complex HLA typing is readily available to physicians trough the health systems, since these assays are routinely performed in diagnostic laboratories for matching donors and patient recipients for organ transplant. Therefore, this pharmacogenetic test is potentially available to practitioners, without the need for a dedicated laboratory. Awareness and proper information on HLA variants associated with antibiotic adverse effects are therefore encouraged, and pharmacoeconomic evaluations to support these assays should be considered.

In conclusion, pharmacogenetic variants associated with interindividual variability in the effect of antibiotics, and in particular in their pharmacokinetics or adverse effects, have been identified, in particular involving genes related to immune mediated effects. While the number of subjects presenting these variants may be limited, given the widespread use of antibiotics and the high number of patients taking these drugs, research and clinical translation of these pharmacogenetic features are of high importance.

## Figures and Tables

**Table 1:** Genetic association with genes related to antibiotic drug pharmacokinetics.

Gene	Polymorphism	Antibiotic	Effect	Reference
*SLC22A8*	rs11568482	Cefotaxime (Cephalosporins)	Lower clearance	[33]
*ABCC2*	rs2273697	Ceftriaxone (Cephalosporins)	Higher drug concentration in CSF	[41]
*ABCG2*	rs13120400		Reduction on drug CSF/plasma ratio	
*ABCC2*	rs717620	Erythromycin (Macrolides)	Increase in drug metabolism	[52]
*SLCO1B1*	rs4149056		Reduction in drug metabolism	[53]
*ABCB1*	2677TT/3435TT	Azithromycin (Macrolides)	Lower C_max_ and higher T_max_	[55]
*UGT1A*	rs8175347	Moxifloxacin (Fluoroquinolones)	Lower clearance	[83]
	rs3755319		Higher clearance	
*ABCB1*	rs2032582		Higher clearance	[83]
*ABCB1*	rs1045642		Higher T_max_	[84]
*SLCO1B1*	rs4149015		Higher AUC_0-24_ and C_max_	[85]
*ABCB1*	1236C > T rs1128503 2677G > T/A rs2032582 3435C > T rs1045642	Daptomycin	Higher AUC_0-24_	[92]
*ABCB1*	rs1045642	Linezolid	Lower clearance	[97]

**Table 2:** MHC class I and II polymorphism associations with adverse reaction to antibiotics.

Gene	HLA Association	Antibiotics	Effect	Reference
*HLA-DRB1*	DRB1*15:01	Amoxicillin clavulanate (Penicillins)	DILI	[16,17,18]
*HLA-DQB1*	DQB1*06:14			[19]
*HLA-DQB1*	rs9274407			[20]
*HLA-DRA*	rs3135388			[20]
*-*	rs2523822			[20]
*HLA-DRB1-HLA-DQB1*	DRB1*15:01-DQB1*06:02			[8,16,17,18]
*HLA-A*	A*30:02			[21]
*HLA-B*	B*18:01			[21]
*HLA-B*	B*57:01	Flucloxacillin (Penicillins)		[24,26]
*HLA-B*	B*57:03			[26]
*HLA-A*	A*32:01	Vancomycin	DRESS	[88]
*HLA-B*	B*35:02	Minocycline	DILI	[99]
*HLA-B*	B*51:01	Clindamycin	Cutaneous reaction	[100]

DILI: drug induced liver injury; DRESS: drug reaction with eosinophilia and systemic symptoms.

## Abbreviations

MHC	Major histocompatibility complex
HLA	Human leukocyte antigen
DILI	Drug induced liver injury
OR	Odds ratios
CI	Confidence index
GWA	Genome wide association
SNP	Single nucleotide polymorphism
MRSA	Methicillin resistant Staphylococcus aureus
MRSE	Methicillin resistant Staphylococcus epidermidis
PXR	Pregnane X receptor
OAT	Organic anion transporter
MRP	Multidrug resistant-associated protein
ABC	ATP binding cassette
OATP	Organic anion transporter polypeptide
SLCO1A2	Solute carrier organic anion transporter family member 1A2
CYP	Cytochrome P450
UGT	Uridine 5′ diphosphate glucuronosyltransferase
AUC0-24	Area under the concentration-time curve from 0 to 24 h
DRESS	Drug reaction with eosinophilia and systemic symptoms

## References

[1] Lesch J. (2007). The First Miracle Drugs: How the sulfa Drugs Transformed Medicine.

[2] Lax E. (2004). The Mold in Dr. Florey’s Coat: The Story of Penicillin Miracle.

[3] Attar M., Lee V.H. (2003). Pharmacogenomic considerations in drug delivery. Pharmacogenomics.

[4] Ho R.H., Kim R.B. (2005). Transporters and drug therapy: Implications for drug disposition and disease. Clin. Pharmacol. Ther..

[5] Pavlos R., Mallal S., Phillips E. (2012). HLA and pharmacogenetics of drug hypersensitivity. Pharmacogenomics.

[6] Romano A., Mondino C., Viola M., Montuschi P. (2003). Immediate allergic reactions to beta-lactams: diagnosis and therapy. Int. J. Immunopathol Pharm..

[7] Kerns D., Shira J.E., Go S., Summers R.J., Schwab J.A., Plunket D.C. (1973). Ampicillin rash in children. Relationship to penicillin allergy and infectious mononucleosis. Am. J. Dis. Child..

[8] Andrade R.J., Lucena M.I., Fernandez M.C., Pelaez G., Pachkoria K., Garcia-Ruiz E., Garcia-Munoz B., Gonzalez-Grande R., Pizarro A., Duran J.A. (2005). Drug-induced liver injury: an analysis of 461 incidences submitted to the Spanish registry over a 10-year period. Gastroenterology.

[9] De Abajo F.J., Montero D., Madurga M., Rodriguez L.A.G. (2004). Acute and clinically relevant drug-induced liver injury: A population based case-control study. Br. J. Clin. Pharmacol..

[10] Chalasani N., Fontana R.J., Bonkovsky H.L., Watkins P.B., Davern T., Serrano J., Yang H., Rochon J., Drug Induced Liver Injury Network (DILIN) (2008). Causes, clinical features, and outcomes from a prospective study of drug-induced liver injury in the United States. Gastroenterology.

[11] Bjornsson E.S., Bergmann O.M., Bjornsson H.K., Kvaran R.B., Olafsson S. (2013). Incidence, presentation, and outcomes in patients with drug-induced liver injury in the general population of Iceland. Gastroenterology.

[12] Andrade R.J., Lucena M.I., Kaplowitz N., Garcia-Munoz B., Borraz Y., Pachkoria K., Garcia-Cortes M., Fernandez M.C., Pelaez G., Rodrigo L. (2006). Outcome of acute idiosyncratic drug-induced liver injury: Long-term follow-up in a hepatotoxicity registry. Hepatology.

[13] Lucena M.I., Andrade R.J., Fernandez M.C., Pachkoria K., Pelaez G., Duran J.A., Villar M., Rodrigo L., Romero-Gomez M., Planas R. (2006). Determinants of the clinical expression of amoxicillin-clavulanate hepatotoxicity: a prospective series from Spain. Hepatology.

[14] Rodriguez L.A.G., Stricker B.H., Zimmerman H.J. (1996). Risk of acute liver injury associated with the combination of amoxicillin and clavulanic acid. Arch. Intern. Med..

[15] Fontana R.J., Shakil A.O., Greenson J.K., Boyd I., Lee W.M. (2005). Acute liver failure due to amoxicillin and amoxicillin/clavulanate. Dig. Dis. Sci..

[16] Hautekeete M.L., Horsmans Y., van Waeyenberge C., Demanet C., Henrion J., Verbist L., Brenard R., Sempoux C., Michielsen P.P., Yap P.S. (1999). HLA association of amoxicillin-clavulanate--induced hepatitis. Gastroenterology.

[17] O’Donohue J., Oien K.A., Donaldson P., Underhill J., Clare M., MacSween R.N., Mills P.R. (2000). Co-amoxiclav jaundice: Clinical and histological features and HLA class II association. Gut.

[18] Donaldson P.T., Daly A.K., Henderson J., Graham J., Pirmohamed M., Bernal W., Day C.P., Aithal G.P. (2010). Human leucocyte antigen class II genotype in susceptibility and resistance to co-amoxiclav-induced liver injury. J. Hepatol..

[19] Andrade R.J., Lucena M.I., Alonso A., Garcia-Cortes M., Garcia-Ruiz E., Benitez R., Fernandez M.C., Pelaez G., Romero M., Corpas R. (2004). HLA class II genotype influences the type of liver injury in drug-induced idiosyncratic liver disease. Hepatology.

[20] Lucena M.I., Molokhia M., Shen Y., Urban T.J., Aithal G.P., Andrade R.J., Day C.P., Ruiz-Cabello F., Donaldson P.T., Stephens C. (2011). Susceptibility to amoxicillin-clavulanate-induced liver injury is influenced by multiple HLA class I and II alleles. Gastroenterology.

[21] Stephens C., Lopez-Nevot M.A., Ruiz-Cabello F., Ulzurrun E., Soriano G., Romero-Gomez M., Moreno-Casares A., Lucena M.I., Andrade R.J. (2013). HLA alleles influence the clinical signature of amoxicillin-clavulanate hepatotoxicity. PLoS ONE.

[22] Cirulli E.T., Nicoletti P., Abramson K., Andrade R.J., Bjornsson E.S., Chalasani N., Fontana R.J., Hallberg P., Li Y.J., Lucena M.I. (2019). A missense variant in PTPN22 is a risk factor for drug-induced liver injury. Gastroenterology.

[23] Peacock S.J., Paterson G.K. (2015). Mechanisms of methicillin resistance in staphylococcus aureus. Annu. Rev. Biochem..

[24] Daly A.K., Donaldson P.T., Bhatnagar P., Shen Y., Pe’er I., Floratos A., Daly M.J., Goldstein D.B., John S., Nelson M.R. (2009). HLA-B*5701 genotype is a major determinant of drug-induced liver injury due to flucloxacillin. Nat. Genet..

[25] Kaplan H.A., Woloski B.M., Hellman M., Jamieson J.C. (1983). Studies on the effect of inflammation on rat liver and serum sialyltransferase. Evidence that inflammation causes release of Gal beta 1 leads to 4GlcNAc alpha 2 leads to 6 sialyltransferase from liver. J. Biol. Chem..

[26] Nicoletti P., Aithal G.P., Chamberlain T.C., Coulthard S., Alshabeeb M., Grove J.I., Andrade R.J., Bjornsson E., Dillon J.F., Hallberg P. (2019). Drug-induced liver injury due to flucloxacillin: Relevance of multiple human leukocyte antigen alleles. Clin. Pharmacol. Ther..

[27] Andrews E., Armstrong M., Tugwood J., Swan D., Glaves P., Pirmohamed M., Aithal G.P., Wright M.C., Day C.P., Daly A.K. (2010). A role for the pregnane X receptor in flucloxacillin-induced liver injury. Hepatology.

[28] Ings R.M., Reeves D.S., White L.O., Bax R.P., Bywater M.J., Holt H.A. (1985). The human pharmacokinetics of cefotaxime and its metabolites and the role of renal tubular secretion on their elimination. J. Pharm. Biopharm..

[29] Ueo H., Motohashi H., Katsura T., Inui K. (2005). Human organic anion transporter hOAT3 is a potent transporter of cephalosporin antibiotics, in comparison with hOAT1. Biochem. Pharmacol..

[30] Uwai Y., Saito H., Inui K. (2002). Rat renal organic anion transporter rOAT1 mediates transport of urinary-excreted cephalosporins, but not of biliary-excreted cefoperazone. Drug Metab. Pharmacokinet..

[31] Jariyawat S., Sekine T., Takeda M., Apiwattanakul N., Kanai Y., Sophasan S., Endou H. (1999). The interaction and transport of beta-lactam antibiotics with the cloned rat renal organic anion transporter 1. J. Pharmacol. Exp. Ther..

[32] Fujita T., Brown C., Carlson E.J., Taylor T., de la Cruz M., Johns S.J., Stryke D., Kawamoto M., Fujita K., Castro R. (2005). Functional analysis of polymorphisms in the organic anion transporter, SLC22A6 (OAT1). Pharm. Genom..

[33] Abecasis G.R., Auton A., Brooks L.D., DePristo M.A., Durbin R.M., Handsaker R.E., Kang H.M., Marth G.T., McVean G.A., 1000 Genome Project Consortium (2012). An integrated map of genetic variation from 1092 human genomes. Nature.

[34] Erdman A.R., Mangravite L.M., Urban T.J., Lagpacan L.L., Castro R.A., de la Cruz M., Chan W., Huang C.C., Johns S.J., Kawamoto M. (2006). The human organic anion transporter 3 (OAT3; SLC22A8): Genetic variation and functional genomics. Am. J. Physiol. Renal. Physiol..

[35] Yee S.W., Nguyen A.N., Brown C., Savic R.M., Zhang Y., Castro R.A., Cropp C.D., Choi J.H., Singh D., Tahara H. (2013). Reduced renal clearance of cefotaxime in asians with a low-frequency polymorphism of OAT3 (SLC22A8). J. Pharm. Sci..

[36] Kato Y., Takahara S., Kato S., Kubo Y., Sai Y., Tamai I., Yabuuchi H., Tsuji A. (2008). Involvement of multidrug resistance-associated protein 2 (Abcc2) in molecular weight-dependent biliary excretion of beta-lactam antibiotics. Drug Metab. Dispos..

[37] Koehn L.M. (2020). ABC efflux transporters at blood-central nervous system barriers and their implications for treating spinal cord disorders. Neural Regen. Res..

[38] Hartz A.M., Bauer B. (2011). ABC transporters in the CNS—An inventory. Curr. Pharm. Biotechnol..

[39] Lee W., Glaeser H., Smith L.H., Roberts R.L., Moeckel G.W., Gervasini G., Leake B.F., Kim R.B. (2005). Polymorphisms in human organic anion-transporting polypeptide 1A2 (OATP1A2): implications for altered drug disposition and central nervous system drug entry. J. Biol. Chem..

[40] Gao B., Hagenbuch B., Kullak-Ublick G.A., Benke D., Aguzzi A., Meier P.J. (2000). Organic anion-transporting polypeptides mediate transport of opioid peptides across blood-brain barrier. J. Pharmacol. Exp. Ther..

[41] Allegra S., Cardellino C.S., Fatiguso G., Cusato J., de Nicolo A., Avataneo V., Bonora S., D’Avolio A., di Perri G., Calcagno A. (2018). Effect of ABCC2 and ABCG2 gene polymorphisms and CSF-to-serum albumin ratio on ceftriaxone plasma and cerebrospinal fluid concentrations. J. Clin. Pharmacol..

[42] Neftel K.A., Hauser S.P., Muller M.R. (1985). Inhibition of granulopoiesis in vivo and in vitro by beta-lactam antibiotics. J. Infect. Dis..

[43] Andres E., Maloisel F. (2008). Idiosyncratic drug-induced agranulocytosis or acute neutropenia. Curr. Opin. Hematol..

[44] Hahn A., Fukuda T., Hahn D., Mizuno T., Frenck R.W., Vinks A.A. (2016). Pharmacokinetics and pharmacogenomics of beta-lactam-induced neutropenia. Pharmacogenomics.

[45] Russel F.G., Koenderink J.B., Masereeuw R. (2008). Multidrug resistance protein 4 (MRP4/ABCC4): a versatile efflux transporter for drugs and signalling molecules. Trends Pharmacol. Sci..

[46] Ci L., Kusuhara H., Adachi M., Schuetz J.D., Takeuchi K., Sugiyama Y. (2007). Involvement of MRP4 (ABCC4) in the luminal efflux of ceftizoxime and cefazolin in the kidney. Mol. Pharmacol..

[47] Vanwert A.L., Bailey R.M., Sweet D.H. (2007). Organic anion transporter 3 (Oat3/Slc22a8) knockout mice exhibit altered clearance and distribution of penicillin G. Am. J. Physiol. Renal. Physiol..

[48] Mc G.J., Bunch R.L., Anderson R.C., Boaz H.E., Flynn E.H., Powell H.M., Smith J.W. (1952). Ilotycin, a new antibiotic. Antibiot. Chemother. (Northfield).

[49] Washington J.A., Wilson W.R. (1985). Erythromycin: A microbial and clinical perspective after 30 years of clinical use (2). Mayo Clin. Proc..

[50] Washington J.A., Wilson W.R. (1985). Erythromycin: A microbial and clinical perspective after 30 years of clinical use (1). Mayo Clin. Proc..

[51] Zuckerman J.M., Qamar F., Bono B.R. (2011). Review of macrolides (azithromycin, clarithromycin), ketolids (telithromycin) and glycylcyclines (tigecycline). Med. Clin. North Am..

[52] Franke R.M., Lancaster C.S., Peer C.J., Gibson A.A., Kosloske A.M., Orwick S.J., Mathijssen R.H., Figg W.D., Baker S.D., Sparreboom A. (2011). Effect of ABCC2 (MRP2) transport function on erythromycin metabolism. Clin. Pharmacol. Ther..

[53] Lancaster C.S., Bruun G.H., Peer C.J., Mikkelsen T.S., Corydon T.J., Gibson A.A., Hu S., Orwick S.J., Mathijssen R.H., Figg W.D. (2012). OATP1B1 polymorphism as a determinant of erythromycin disposition. Clin. Pharmacol. Ther..

[54] Sugie M., Asakura E., Zhao Y.L., Torita S., Nadai M., Baba K., Kitaichi K., Takagi K., Takagi K., Hasegawa T. (2004). Possible involvement of the drug transporters P glycoprotein and multidrug resistance-associated protein Mrp2 in disposition of azithromycin. Antimicrob. Agents Chemother..

[55] He X.J., Zhao L.M., Qiu F., Sun Y.X., Li-Ling J. (2009). Influence of ABCB1 gene polymorphisms on the pharmacokinetics of azithromycin among healthy Chinese Han ethnic subjects. Pharmacol. Rep..

[56] Becker B., Cooper M.A. (2013). Aminoglycoside antibiotics in the 21st century. ACS Chem. Biol..

[57] Guthrie O.W. (2008). Aminoglycoside induced ototoxicity. Toxicology.

[58] Selimoglu E. (2007). Aminoglycoside-induced ototoxicity. Curr. Pharm. Des..

[59] Guan M.X. (2011). Mitochondrial 12S rRNA mutations associated with aminoglycoside ototoxicity. Mitochondrion.

[60] Prezant T.R., Agapian J.V., Bohlman M.C., Bu X., Oztas S., Qiu W.Q., Arnos K.S., Cortopassi G.A., Jaber L., Rotter J.I. (1993). Mitochondrial ribosomal RNA mutation associated with both antibiotic-induced and non-syndromic deafness. Nat. Genet..

[61] Estivill X., Govea N., Barcelo E., Badenas C., Romero E., Moral L., Scozzri R., D’Urbano L., Zeviani M., Torroni A. (1998). Familial progressive sensorineural deafness is mainly due to the mtDNA A1555G mutation and is enhanced by treatment of aminoglycosides. Am. J. Hum. Genet..

[62] Bitner-Glindzicz M., Pembrey M., Duncan A., Heron J., Ring S.M., Hall A., Rahman S. (2009). Prevalence of mitochondrial 1555A-->G mutation in European children. N. Engl. J. Med..

[63] Vandebona H., Mitchell P., Manwaring N., Griffiths K., Gopinath B., Wang J.J., Sue C.M. (2009). Prevalence of mitochondrial 1555A-->G mutation in adults of European descent. N. Engl. J. Med..

[64] Zhao H., Li R., Wang Q., Yan Q., Deng J.H., Han D., Bai Y., Young W.Y., Guan M.X. (2004). Maternally inherited aminoglycoside-induced and nonsyndromic deafness is associated with the novel C1494T mutation in the mitochondrial 12S rRNA gene in a large Chinese family. Am. J. Hum. Genet..

[65] FDA Reinforces Safety Information about Serious Low Blood Sugar Levels and Mental Health Side Effects With Fluoroquinolone Antibiotics. https://www.fda.gov/drugs/drug-safety-and-availability/fda-reinforces-safety-information-about-serious-low-blood-sugar-levels-and-mental-health-side.

[66] Disabling and Potentially Permanent Side Effects Lead to Suspension or Restrictions of Quinolone and Fluoroquinolone Antibiotics. https://www.ema.europa.eu/en/documents/referral/quinolone-fluoroquinolone-article-31-referral-disabling-potentially-permanent-side-effects-lead_en.pdf.

[67] Mehlhorn A.J., Brown D.A. (2007). Safety concerns with fluoroquinolones. Ann. Pharmacother..

[68] Leslie E.M., Deeley R.G., Cole S.P. (2005). Multidrug resistance proteins: role of P-glycoprotein, MRP1, MRP2, and BCRP (ABCG2) in tissue defense. Toxicol. Appl. Pharmacol..

[69] Alvarez A.I., Perez M., Prieto J.G., Molina A.J., Real R., Merino G. (2008). Fluoroquinolone efflux mediated by ABC transporters. J. Pharm. Sci..

[70] Ooie T., Terasaki T., Suzuki H., Sugiyama Y. (1997). Quantitative brain microdialysis study on the mechanism of quinolones distribution in the central nervous system. Drug Metab. Dispos..

[71] Ooie T., Terasaki T., Suzuki H., Sugiyama Y. (1997). Kinetic evidence for active efflux transport across the blood-brain barrier of quinolone antibiotics. J. Pharmacol. Exp. Ther..

[72] De Lange E.C., Marchand S., van den Berg D., van der Sandt I.C., de Boer A.G., Delon A., Bouquet S., Couet W. (2000). In vitro and in vivo investigations on fluoroquinolones; effects of the P-glycoprotein efflux transporter on brain distribution of sparfloxacin. Eur. J. Pharm. Sci..

[73] Gervasoni C., Cattaneo D., Falvella F.S., Vitiello P., Cheli S., Milazzo L., Clementi E., Riva A. (2013). Levofloxacin-induced seizures in a patient without predisposing risk factors: the impact of pharmacogenetics. Eur. J. Clin. Pharmacol..

[74] Kroetz D.L., Pauli-Magnus C., Hodges L.M., Huang C.C., Kawamoto M., Johns S.J., Stryke D., Ferrin T.E., DeYoung J., Taylor T. (2003). Sequence diversity and haplotype structure in the human ABCB1 (MDR1, multidrug resistance transporter) gene. Pharmacogenetics.

[75] Franke R.M., Scherkenbach L.A., Sparreboom A. (2009). Pharmacogenetics of the organic anion transporting polypeptide 1A2. Pharmacogenomics.

[76] Maeda T., Takahashi K., Ohtsu N., Oguma T., Ohnishi T., Atsumi R., Tamai I. (2007). Identification of influx transporter for the quinolone antibacterial agent levofloxacin. Mol. Pharm..

[77] MacGowan A.P. (1999). Moxifloxacin (Bay 12-8039): a new methoxy quinolone antibacterial. Expert Opin. Investig. Drugs.

[78] WHO (2019). Global Tuberculosis Report. https://www.who.int/tb/publications/global_report/en/.

[79] Naidoo A., Naidoo K., McIlleron H., Essack S., Padayatchi N. (2017). A review of moxifloxacin for the treatment of drug-susceptible tuberculosis. J. Clin. Pharmacol..

[80] Pranger A.D., Kosterink J.G., van Altena R., Aarnoutse R.E., van der Werf T.S., Uges D.R., Alffenaar J.W. (2011). Limited-sampling strategies for therapeutic drug monitoring of moxifloxacin in patients with tuberculosis. Ther. Drug Monit..

[81] Barbarino J.M., Haidar C.E., Klein T.E., Altman R.B. (2014). PharmGKB summary: Very important pharmacogene information for UGT1A1. Pharmacogenet. Genomics.

[82] Stingl J.C., Bartels H., Viviani R., Lehmann M.L., Brockmoller J. (2014). Relevance of UDP-glucuronosyltransferase polymorphisms for drug dosing: A quantitative systematic review. Pharmacol. Ther..

[83] Naidoo A., Ramsuran V., Chirehwa M., Denti P., McIlleron H., Naidoo K., Yende-Zuma N., Singh R., Ngcapu S., Chaudhry M. (2018). Effect of genetic variation in UGT1A and ABCB1 on moxifloxacin pharmacokinetics in South African patients with tuberculosis. Pharmacogenomics.

[84] Weiner M., Burman W., Luo C.C., Peloquin C.A., Engle M., Goldberg S., Agarwal V., Vernon A. (2007). Effects of rifampin and multidrug resistance gene polymorphism on concentrations of moxifloxacin. Antimicrob. Agents Chemother..

[85] Weiner M., Gelfond J., Johnson-Pais T.L., Engle M., Peloquin C.A., Johnson J.L., Sizemore E.E., Mac Kenzie W.R. (2018). Elevated plasma moxifloxacin concentrations and SLCO1B1 g-11187G>A polymorphism in adults with pulmonary tuberculosis. Antimicrob. Agents Chemother..

[86] Malik M., Hnatkova K., Schmidt A., Smetana P. (2009). Electrocardiographic QTc changes due to moxifloxacin infusion. J. Clin. Pharmacol..

[87] Levine D.P. (2006). Vancomycin: A history. Clin. Infect. Dis..

[88] Konvinse K.C., Trubiano J.A., Pavlos R., James I., Shaffer C.M., Bejan C.A., Schutte R.J., Ostrov D.A., Pilkinton M.A., Rosenbach M. (2019). HLA-A*32:01 is strongly associated with vancomycin-induced drug reaction with eosinophilia and systemic symptoms. J. Allergy Clin. Immunol..

[89] Van Driest S.L., McGregor T.L., Edwards D.R.V., Saville B.R., Kitchner T.E., Hebbring S.J., Brilliant M., Jouni H., Kullo I.J., Creech C.B. (2015). Genome-wide association study of serum creatinine levels during vancomycin therapy. PLoS ONE.

[90] Shoemaker D.M., Simou J., Roland W.E. (2006). A review of daptomycin for injection (Cubicin) in the treatment of complicated skin and skin structure infections. Ther. Clin. Risk Manag..

[91] Lemaire S., van Bambeke F., Mingeot-Leclercq M.P., Tulkens P.M. (2007). Modulation of the cellular accumulation and intracellular activity of daptomycin towards phagocytized Staphylococcus aureus by the P-glycoprotein (MDR1) efflux transporter in human THP-1 macrophages and madin-darby canine kidney cells. Antimicrob. Agents Chemother..

[92] Baietto L., D’Avolio A., Cusato J., Pace S., Calcagno A., Motta I., Corcione S., di Perri G., de Rosa F.G. (2015). Effect of SNPs in human ABCB1 on daptomycin pharmacokinetics in Caucasian patients. J. Antimicrob. Chemother..

[93] Rivera A.M., Boucher H.W. (2011). Current concepts in antimicrobial therapy against select gram-positive organisms: methicillin-resistant *Staphylococcus aureus*, penicillin-resistant pneumococci, and vancomycin-resistant enterococci. Mayo Clin. Proc..

[94] Bolhuis M.S., Tiberi S., Sotgiu G., de Lorenzo S., Kosterink J.G., van der Werf T.S., Migliori G.B., Alffenaar J.W. (2015). Linezolid tolerability in multidrug-resistant tuberculosis: A retrospective study. Eur. Respir. J..

[95] Zoller M., Maier B., Hornuss C., Neugebauer C., Dobbeler G., Nagel D., Holdt L.M., Bruegel M., Weig T., Grabein B. (2014). Variability of linezolid concentrations after standard dosing in critically ill patients: a prospective observational study. Crit. Care.

[96] Dong H., Xie J., Wang T., Chen L., Zeng X., Sun J., Wang X., Dong Y. (2016). Pharmacokinetic/pharmacodynamic evaluation of linezolid for the treatment of staphylococcal infections in critically ill patients. Int. J. Antimicrob. Agents.

[97] Allegra S., di Paolo A., Cusato J., Fatiguso G., Arrigoni E., Danesi R., Corcione S., D’Avolio A. (2018). A common mdr1 gene polymorphism is associated with changes in linezolid clearance. Ther. Drug Monit..

[98] Bjornsson E., Talwalkar J., Treeprasertsuk S., Kamath P.S., Takahashi N., Sanderson S., Neuhauser M., Lindor K. (2010). Drug-induced autoimmune hepatitis: clinical characteristics and prognosis. Hepatology.

[99] Urban T.J., Nicoletti P., Chalasani N., Serrano J., Stolz A., Daly A.K., Aithal G.P., Dillon J., Navarro V., Odin J. (2017). Minocycline hepatotoxicity: Clinical characterization and identification of HLA-B *35:02 as a risk factor. J. Hepatol..

[100] Yang Y., Chen S., Yang F., Zhang L., Alterovitz G., Zhu H., Xuan J., Yang X., Luo H., Mu J. (2017). HLA-B*51:01 is strongly associated with clindamycin-related cutaneous adverse drug reactions. Pharm. J..

